# Application and Impact of Antiviral Therapy for Patients with HBV-Related Hepatocellular Carcinoma Receiving Sorafenib and Lenvatinib Treatment

**DOI:** 10.3390/v14112355

**Published:** 2022-10-26

**Authors:** I-Cheng Lee, Pei-Chang Lee, Yee Chao, Chen-Ta Chi, Chi-Jung Wu, Yi-Ping Hung, Chien-Wei Su, Ming-Chih Hou, Yi-Hsiang Huang

**Affiliations:** 1 Division of Gastroenterology and Hepatology, Department of Medicine, Taipei Veterans General Hospital, Taipei 112, Taiwan; 2 School of Medicine, National Yang Ming Chiao Tung University, Taipei 112, Taiwan; 3 Cancer Center, Taipei Veterans General Hospital, Taipei 112, Taiwan; 4 Institute of Clinical Medicine, National Yang Ming Chiao Tung University, Taipei 112, Taiwan

**Keywords:** hepatitis B virus, hepatocellular carcinoma, tyrosine kinase inhibitor, antiviral therapy, sorafenib, lenvatinib, entecavir, tenofovir

## Abstract

Overall survival (OS) in patients with advanced hepatocellular carcinoma (HCC) has improved in the era of multi-line sequential therapy. The application of antiviral therapy and its impact on survival for patients with HBV-related HCC needs to be reassessed. The aim of this study was to evaluate the application and impact of antiviral therapy on survival for patients with HBV-related HCC receiving tyrosine kinase inhibitor (TKI) therapy. Patients with advanced HBV-related HCC treated with sorafenib or lenvatinib as first-line therapy with (*n* = 377) and without (*n* = 182) nucleos(t)ide analogue (NUC) therapy were retrospectively enrolled. Prognostic factors of OS were evaluated. Secular trends in the increased application of NUC therapy and improved survival were observed in the last decade. The HBV reactivation rate in patients without NUC therapy was 6.6%. By multivariate analysis, baseline low HBV viral load, achieving undetectable HBV DNA after TKI therapy, and ability to receive second-line therapy were found to be independent predictors of OS. In subgroup patients with NUC therapy, starting NUC before TKI was associated with a better OS. In conclusion, the application of antiviral therapy for patients with HBV-related HCC receiving TKI therapy has increased over time. Achieving complete virological suppression may contribute to a better OS in patients with advanced HBV-related HCC.

## 1. Introduction

Hepatocellular carcinoma (HCC) is the third leading cause of cancer-related deaths in the world, and chronic hepatitis B virus (HBV) infection is the major cause of HCC, especially in Asia [1]. HBV viral activity has a negative impact on the outcomes in patients with HBV-related HCC, and antiviral therapy has been shown to decrease the risk of HCC recurrence and improve survival in patients with early-stage HCC after curative treatment [2,3,4,5]. In patients with advanced-stage HCC, atezolizumab plus bevacizumab is currently the recommended first-line treatment [6,7], while tyrosine kinase inhibitors (TKI) including sorafenib [8,9] or lenvatinib [10] remain the first-line treatment options in cases where atezolizumab plus bevacizumab is contraindicated.

In patients receiving sorafenib for advanced-stage HCC, previous studies showed that high HBV viral load was associated with a poorer overall survival (OS), whereas antiviral therapy might provide a survival benefit [11,12,13]. Liver function decline is frequently encountered during TKI therapy and was associated with worse survival outcomes [14,15]. The risk of liver function deterioration during TKI therapy might be higher in patients with a high viral load but no antiviral therapy. Furthermore, HBV reactivation is also a specific concern in HBV-infected patients receiving anti-cancer treatment [16,17]. HBV reactivation could also develop in patients with HBV-related HCC receiving TKI therapy, but only a few studies with limited case numbers have been reported [18].

Several second-line treatment options have become available for advanced-stage HCC in the past few years, including regorafenib, cabozantinib, ramucirumab, and immune checkpoint inhibitors (ICIs) [19]. Nevertheless, patients with deteriorated liver function at the time of disease progression may have less chance to receive second-line HCC treatment [20,21]. The OS in patients with advanced HCC gradually improved in the era of multi-line sequential therapy. The application of antiviral therapy and its impact on survival for patients with HBV-related HCC needs to be reassessed. The aim of this study was to evaluate the application and impact of antiviral therapy on survival for patients with HBV-related HCC receiving TKI therapy.

## 2. Material and Methods

### 2.1. Patients

**Figure 1:**
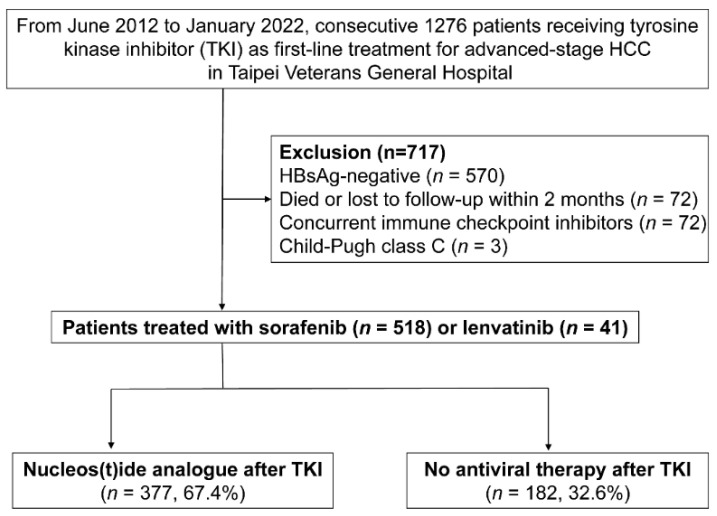
Screening, enrollment, and grouping of patients.

From June 2012 to January 2022, we retrospectively screened 1276 patients who received sorafenib or lenvatinib as first-line therapy for unresectable HBV-related HCC in Taipei Veterans General Hospital (Figure 1). Patients were included if they fulfilled the following criteria: (1) age ≥ 20 years; (2) diagnosis of HCC according to the criteria of the American Association for the Study of Liver Diseases (AASLD) guidelines [22]; and (3) Barcelona Clinic Liver Cancer (BCLC) stage C or stage B refractory to transarterial chemoembolization (TACE). The exclusion criteria were as follows: (1) HBsAg-negative (*n* = 570); (2) died or lost to follow-up within 2 months after starting (*n* = 72); (3) receiving concurrent immunotherapy during TKI therapy (*n* = 72); (4) Child–Pugh class C (*n* = 3).

This study adhered to the guidelines Declaration of Helsinki and gained the consent of the Institutional Review Board in Taipei Veterans General Hospital (IRB number: 2022-08-015AC). The Institutional Review Board waived the need for written in-formed consent due to the retrospective nature of the study.

The standard regimen of sorafenib was 400 mg twice daily [20,23,24], while the daily dose of lenvatinib was 12 mg for patients with body weight  ≥ 60 kg and 8 mg for patients with body weight < 60 kg [25]. The dosage of sorafenib or lenvatinib was modified upon the development of adverse events. Treatments of sorafenib or lenvatinib were continued until the occurrence of radiologic progression, death, or unacceptable adverse events.

### 2.2. Patient Assessment

Tumor measurements were performed at screening and every 2 months during treatment by contrast-enhanced computed tomography (CECT) or magnetic resonance imaging (MRI), which complied with the regulations of the National Health Insurance Administration, Taiwan. Patients visited the clinic every 2 to 4 weeks for evaluation of liver function and assessment of adverse events [20,23,24]. Detailed demographic profile, tumor characteristics, and biochemistry data at baseline and during follow-up were recorded. These data included age, gender, serum HBV DNA, HBsAg, HBeAg, anti-hepatitis C virus antibody, type of nucleos(t)ide analogues (NUCs) for HBV, BCLC stage, macrovascular invasion, extrahepatic metastasis, serum creatinine, albumin, total bilirubin, aspartate aminotransferase (AST), alanine aminotransferase (ALT), alpha-fetoprotein (AFP) levels, and platelet count. The ALBI score and grade were calculated as previously described [26,27]. HBV DNA levels were measured by the Roche Cobas Taqman HBV DNA assay with detection limit of 20 IU/mL (Roche Diagnostics, Switzerland).

### 2.3. Outcome Assessment

We used the modified Response Evaluation Criteria in Solid Tumors (mRECIST) criteria to assess radiologic response [24,28]. Disease progression was defined as an increase of at least 20% in the sum of the diameters of viable target lesions evaluated by CECT or MRI every 2 months during TKI treatment. Progression-free survival (PFS) was defined as the time interval between the day of the starting TKI treatment and disease progression. Overall survival (OS) was defined as the time interval between the day of the starting TKI treatment and death.

Patients who started antiviral therapy within 2 months of starting TKI therapy were classified as the NUC therapy group, whereas patients without antiviral therapy, or who started antiviral therapy after 2 months of starting TKI therapy, were classified as the non-antiviral therapy group. The virological response was defined as achieving undetectable HBV DNA after NUC therapy [17]. HBV reactivation was defined according to the AASLD criteria [29].

### 2.4. Statistical Analysis

IBM SPSS Statistics for Windows, Version 22 (IBM, Armonk, NY), was used to perform all statistical analyses. Values were expressed as mean ± standard deviation (SD) or median (interquartile range, IQR) when appropriate. Continuous variables were compared by Mann–Whitney U test. Categorical variables were compared by Pearson chi-square analysis. The survival rate was estimated by the Kaplan–Meier method. The survival curve between patient groups was compared by the log-rank test. The Cox proportional hazards model was used for analysis of survival factors. Factors that achieved *p* < 0.1 by univariate analysis were subsequently included in the multivariate analysis. A two-tailed *p*-value < 0.05 was considered as statistically significant.

## 3. Results

### 3.1. Patient Characteristics

This study enrolled a total of 559 patients, including 377 patients in the NUC therapy group and 182 patients in the non-antiviral therapy group (Figure 1). The baseline characteristics of the enrolled patients are shown in Table 1. Most of the patients were in BCLC stage C and Child–Pugh class A. Patients in the NUC therapy group had significant lower serum HBV DNA levels, a higher proportion of patients with undetectable HBV DNA, and lower AFP levels. The BCLC stage, status of portal vein invasion, extrahepatic metastasis, Child–Pugh class, and ALBI grade were comparable between the two groups. In patients with antiviral therapy, the majority of them received NUCs with a high genetic barrier (entecavir, tenofovir disoproxil fumarate, tenofovir alafenamide), and started antiviral therapy before the use of TKI (83.3%).

### 3.2. Main Outcomes in Patients with and without NUC Therapy

The median follow-up period was 14.1 and 11.9 months in the NUC therapy and non-antiviral therapy groups, respectively (*p* = 0.442). The median duration of sorafenic/lenvatinib treatment was 86 and 73.5 days in those with and without NUC therapy, respectively (*p* = 0.156). The major outcomes in patients with and without NUC therapy are shown in Table 2. The PFSs were not significantly different in patients with and without antiviral therapy (2.6 vs. 2.4 months, *p* = 0.914), whereas the OS was significantly longer in the NUC therapy group (9.2 vs. 8.1 months, *p* = 0.017). Patients in the NUC therapy group had a significantly higher chance of receiving second-line systemic therapy (41.4% vs. 30.8%, *p* = 0.020). The virological response rate was 82% in patients with NUC therapy. In patients without NUC therapy, 12 (6.6%) cases developed HBV reactivation, and 2 (16.7%) of them died of hepatic decompensation despite immediate NUC therapy after HBV reactivation.

### 3.3. Secular Trends in Overall Survival and NUC Therapy Uptake from 2012–2022

**Figure 2:**
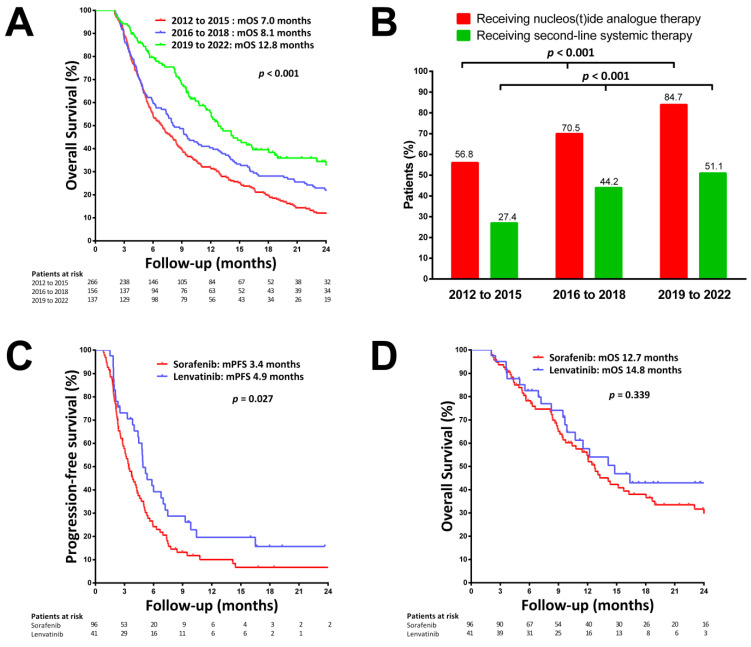
Secular trends in overall survival (OS) and nucleos(t)ide analogue (NUC) therapy uptake from 2012 to 2022. (**A**) Secular trends in OS from 2012 to 2022. (**B**) Proportion of patients receiving NUC therapy and second-line systemic therapy from 2012 to 2022. (**C**) Progression-free survival (PFS) in patients receiving lenvatinib or sorafenib from 2019 to 2022. (**D**) OS in patients receiving lenvatinib or sorafenib during 2019–2022.

We classified patients into three different periods of starting TKI therapy: 2012–2015 (*n* = 266), 2016–2018 (*n* = 156), and 2019–2022 (*n* = 137). As shown in Figure 2A, the OS gradually improved after 7.0 months in patients who started TKI during 2012–2015 to 8.1 months during 2016–2018 and 12.7 months during 2019–2022 (*p* < 0.001). There was a significant trend in increased application of NUC therapy from 56.8% during 2012–2015, to 70.5% during 2016–2018, and to 84.7% during 2019–2022 (*p* < 0.001, Figure 2B). There was also a significant trend in increased application of second-line systemic therapy from 27.4% during 2012–2015, to 44.2% during 2016–2018, and to 51.1% during 2019–2022 (*p* < 0.001, Figure 2B).

Because lenvatinib was introduced in Taiwan in 2019, we compared the PFS and OS between patients receiving lenvatinib and sorafenib during 2019–2022. The median PFS was significantly longer in patients with lenvatinib treatment (4.9 vs. 3.4 months, Figure 2C), while the OS was comparable between patients treated with lenvatinib and sorafenib (14.8 vs. 12.7 months, *p* = 0.339, Figure 2D).

### 3.4. Factors Associated with Overall Survival

**Figure 3:**
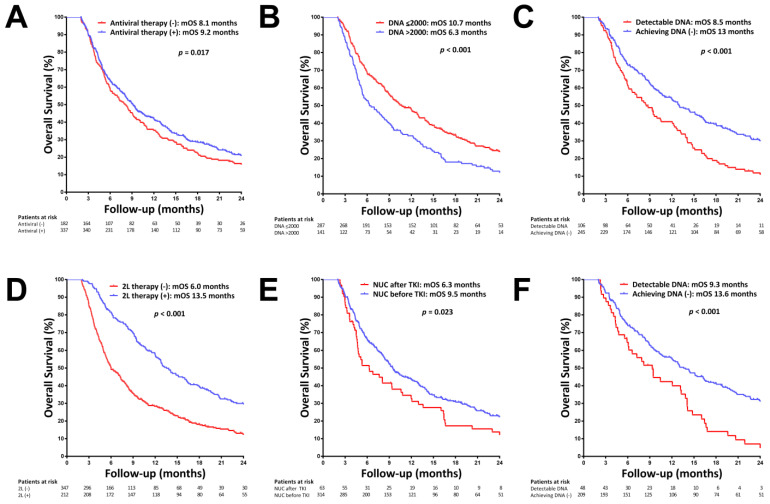
Kaplan–Meier curves of overall survival (OS) in patients with HBV-related HCC. (**A**) OS in patients with and without nucleos(t)ide analogue (NUC) therapy. (**B**) OS stratified by HBV DNA level. (**C**) OS in patients with and without achieving undetectable HBV DNA. (**D**) OS in patients with and without second-line (2L) therapy. (**E**) OS in subgroup patients with NUC therapy who started NUC before and after TKI treatment. (**F**) OS in subgroup patients with NUC therapy with and without achieving undetectable HBV DNA.

By univariate analysis, antiviral therapy (Figure 3A), BCLC stage, portal vein invasion, extrahepatic metastasis, ALBI grade, serum AFP, HBV DNA, ALT, AST levels, achieving undetectable HBV DNA, and ability to receive second-line therapy were significantly associated with OS. By multivariate analysis, BCLC stage (hazard ratio (HR) = 1.971, *p* = 0.002), baseline AFP > 400 ng/mL (HR = 1.374, *p* = 0.004), HBV DNA > 2000 IU/mL (HR = 1.309, *p* = 0.017, Figure 3B), and ALBI grade 2–3 (HR = 1.609, *p* < 0.001) were baseline predictors of OS, while achieving undetectable HBV DNA (HR = 0.609, *p* = 0.002, Figure 3C) and ability to receive second-line therapy (*p* = 0.630, *p* = 0.001, Figure 3D) were on-treatment predictors of OS (Table 3).

In subgroup patients with NUC therapy, BCLC stage (HR = 1.939, *p* = 0.004), baseline AFP > 400 ng/mL (HR = 1.439, *p* = 0.004), NUC therapy started before the use of TKI (HR = 1.309, *p* = 0.017, Figure 3E), and ALBI grade 2–3 (HR = 1.609, *p* < 0.001) were baseline predictors of OS, while achieving undetectable HBV DNA (HR = 0.609, *p* = 0.002, Figure 3F) and ability to receive second-line therapy (*p* = 0.630, *p* = 0.001) were on-treatment predictors of OS (Table 4).

## 4. Discussion

In this study, we evaluated the secular trends in the application of NUC therapy and its impact on survival in patients with advanced HCC receiving TKI. We showed an increased application of antiviral therapy and improved OS over time in the last decade. In particular, achieving complete HBV suppression was identified as an important factor associated with improved survival.

Our data showed that the median OS in patients with advanced HCC receiving TKI therapy significantly improved from 7 months during 2012–2015 to 12.8 months during 2019–2022. The application of NUC therapy and second-line systemic therapy also significantly increased during 2019–2022. A previous study showed that physicians’ experience in managing adverse events with tailored sorafenib dosing has improved over time, which has led to increased sorefenib treatment duration and prolonged survival of HCC patients [30]. A recent study from Hong Kong showed that the secular trend of NUC therapy uptake gradually increased in recent years, which may also have contributed to the improved survival in patients with HBV-related HCC [31]. Lenvatinib and regorafenib were reimbursed by the national health insurance program in Taiwan after 2019. Other treatment options, such as immunotherapy, cabozantinib, and ramicirumab, were also available options as second-line therapy in recent years [21,25]. Consistent with the REFLECT trial and recent real-world studies [10,32,33], our data showed that the median OS was comparable between patients receiving lenvatinib and sorafenib during 2019–2022, even though numerically longer by Lenvatinib, while the median PFS was significantly longer in patients with lenvatinib treatment. Our data also showed that patients with NUC therapy were associated with a higher chance of receiving second-line systemic therapy. Our recent study showed that patients who were able to receive regorafenib after sorafenib failure had a median OS of 13.1 months after starting regorafenib treatment [21]. These data suggest that the application of NUC therapy and multi-line sequential therapy may prolong the survival of patients with advanced HBV-related HCC.

The virological response rate after NUC therapy was 81.3%, which was lower than the response rate of NUC prophylaxis for cancer patients undergoing chemotherapy [16,17]. Since patients with advanced HCC generally had higher baseline viral load and shorter OS as compared to cancer patients undergoing chemotherapy, the virological response might not be achieved in the short term after TKI therapy. There were only a few studies that reported the risk of HBV reactivation during TKI therapy for patients with HCC, and a recent systemic review showed that the pooled rate of HBV reactivation was 6.2% after sorafenib therapy [11,18]. In this study, we reported an HBV reactivation rate of 6.6% in patients without antiviral therapy, suggesting a moderate risk of HBV reactivation after TKI therapy for HBV-related HCC.

BCLC stage, ALBI grade, AFP level, and high HBV viral load were identified as independent baseline predictors of OS, while achieving undetectable HBV DNA and the ability to receive second-line therapy were on-treatment predictors of OS. Tumor factors and liver function reserve are well-known prognostic factors of patients with HCC [23,27,34]. HBV viral factors may also have an important impact on the prognosis of HBV-related HCC [3,11,35]. A recent study showed that patients with well-controlled viremia had a significantly better OS after sorafenib treatment [36]. Consistent with this study, our data showed that baseline low HBV viral load or undetectable HBV DNA was associated with a significantly better OS. Furthermore, achieving undetectable HBV DNA during TKI therapy was also an independent predictor of OS. In subgroup patients with NUC therapy, patients who had already received NUC treatment before starting TKI had significantly better OS than those who started NUC treatment after TKI. Patients who started NUC therapy earlier may achieve complete viral suppression earlier, which may lead to better survival after TKI treatment.

There are some limitations in this study. First, this is a retrospective study from a single tertiary medical center. Some patients lacked HBV DNA data at baseline and after TKI therapy. Especially patients with shorter OS may not have the chance to check virological response during follow-up. Second, the case number among patients receiving lenvatinib treatment was relatively small. A significant proportion of patients receiving lenvatinib in our institution had concurrent pembrolizumab use and were excluded from analysis [25]. The impact of NUC therapy in patients with TKI plus immune checkpoint inhibitor therapy also warrants further study. Third, the impact of quantitative HBsAg level and HBV genotype could not be assessed in this study. In Taiwan, the majority of CHB patients were infected with HBV genotype B or C [37,38]. Whether HBsAg level and HBV genotype had a prognostic impact in patients with HCC receiving TKI therapy needs further investigation.

In conclusion, the application of NUC therapy for patients with HBV-related HCC receiving TKI therapy has increased over time. Patients receiving NUC therapy may have a higher chance of achieving complete virological suppression and a higher chance of receiving second-line systemic therapy after first-line TKI failure, which may contribute to a better OS in patients with advanced HBV-related HCC.

## Figures and Tables

**Table 1:** Baseline characteristics of the 559 patients with HBV-related HCC with and without nucleos(t)ide analogue (NUC) therapy.

	With NUC Therapy (*n* = 377)	Without NUC Therapy (*n* = 182)	*p*
Age (years)	62.2 ± 12.5	63.3 ± 13.2	0.318
Sex (male), *n* (%)	319 (84.6)	158 (86.8)	0.575
HCC treatment: Sorafenib/lenvatinib, *n* (%)	340/37 (90.2/9.8)	178/4 (97.8/2.2)	0.002
BCLC stage B/C, *n* (%)	331 (87.8)	176 (96.7)	0.001
Portal vein invasion, *n* (%)	210 (55.7)	111 (61)	0.274
Vp4	62 (16.4)	26 (14.3)	0.594
Extrahepatic metastasis, *n* (%)	197 (52.3)	106 (58.2)	0.215
Child-Pugh class A/B, *n* (%)	38 (10.1)	25 (13.7)	0.255
ALBI score	−2.47 ± 0.49	−2.48 ± 0.44	0.896
ALBI grade 1/2/3, *n* (%)	160/211/6 (42.4/56/1.6)	75/105/2 (41.2/57.7/1.1)	0.875
HBV DNA (Log IU/mL) *	2.72 ± 2	3.08 ± 1.99	0.013
Undetectable HBV DNA, *n* (%) *	161 (47.9)	27 (29.3)	0.002
HBV DNA < 2000 IU/mL, *n* (%) *	230 (68.5)	57 (62)	0.294
NUC type: LMV/ADV/ETV/TDF/TAF, *n* (%)	6/3/305/31/32 (1.6/0.8/80.9/8.2/8.5)	-	
NUC therapy before TKI, *n* (%)	314 (83.3)	-	
Bilirubin (mg/dL)	1.04 ± 1.24	0.93 ± 0.51	0.387
Albumin (g/dL)	3.80 ± 0.51	3.80 ± 0.46	0.695
ALT (U/L)	52 ± 48	52 ± 50	0.956
AST (U/L)	72 ± 68	68 ± 56	0.766
Platelet (10^9^/L)	173 ± 95	179 ± 100	0.496
AFP (ng/mL)	269 (16–4766)	1153 (19–8682)	0.028

LMV, lamivudine; ADV, adefovir; ETV, entecavir; TDF, tenofovir disoproxil fumarate; TAF, tenofovir alafenamide; NUC, TKI, tyrosine kinase inhibitor. * A total of 428 (76.6%) cases had available baseline HBV DNA data.

**Table 2:** Major outcomes patients with HBV-related HCC with and without nucleos(t)ide analogue (NUC) therapy.

	With NUC Therapy (*n* = 377)	Without NUC Therapy (*n* = 182)	*p*
Follow-up period (months)	14.1 (2–103.3)	11.9 (2–61.2)	0.442
Duration of Sorafenic/Lenvatinib treatment (days)	86 (59–145)	73.5 (56–131.5)	0.156
Disease progression, *n* (%)	159 (87.4)	332 (88.1)	0.921
Progression-free survival (months)	2.6 (2.2–2.9)	2.4 (2.0–2.8)	0.914
Second-line systemic therapy, *n* (%)	156 (41.4)	56 (30.8)	0.020
Death, *n* (%)	306 (81.2)	170 (93.4)	<0.001
Overall survival (months)	9.2 (8.0–10.4)	8.1 (6.4–9.7)	0.017
Virological response, *n* (%) *	209 (81.3)	-	-
HBV reactivation, *n* (%)	-	12 (6.6)	-
HBV reactivation-related death, *n* (%)	-	2 (16.7)	-

* A total of 257 (68.2%) cases had available follow-up HBV DNA data.

**Table 3:** Univariate and multivariate analyses of factors associated with overall survival.

		Univariate		Multivariate Model I ^†^		Multivariate Model II ^†^	
		HR (95% CI)	*p*	HR (95% CI)	*p*	HR (95% CI)	*p*
Baseline factors							
Age (years)	>60 vs. ≤60	0.889 (0.741–1.066)	0.205				
Sex	Female vs. male	1.035 (0.802–1.337)	0.792				
BCLC stage	C vs. B	2.678 (1.803–3.979)	<0.001	1.971 (1.273–3.052)	0.002	1.695 (1.077–2.666)	0.023
Portal vein invasion	Yes vs. no	1.633 (1.356–1.967)	<0.001		NS		NS
Vp4	Yes vs. no	1.412 (1.106–1.804)	0.006		NS		NS
Extrahepatic metastasis	Yes vs. no	1.128 (0.941–1.351)	0.193				
AFP (ng/mL)	>400 vs. ≤400	1.659 (1.384–1.988)	<0.001	1.374 (1.374–1.109)	0.004		NS
HBV DNA (IU/mL)	>20 vs. ≤20	1.442 (1.168–1.779)	0.001		NS		NS
	>2000 vs. ≤2000	1.486 (1.195–1.847)	<0.001	1.309 (1.049–1.634)	0.017		NS
NUC therapy	Yes vs. no	0.796 (0.660–0.961)	0.018		NS		NS
ALBI grade	2–3 vs. 1	1.874 (1.554–2.260)	<0.001	1.609 (1.298–1.996)	<0.001	1.688 (1.285–2.217)	<0.001
ALT (U/L)	>40 vs. ≤40	1.280 (1.069–1.533)	0.007		NS		NS
AST (U/L)	>40 vs. ≤40	1.562 (1.291–1.889)	<0.001		NS		NS
Platelet count (10^9^/L)	>150 vs. ≤150	0.971 (0.811–1.163)	0.752				
On-treatment factors							
Achieving undetectable HBV DNA	Yes vs. no	0.533 (0.432–0.709)	<0.001			0.609 (0.442–0.839)	0.002
Second-line therapy	Yes vs. no	0.523 (0.433–0.632)	<0.001			0.630 (0.481–0.824)	0.001

Abbreviations: CI, confidence interval; HR, hazard ratio; NS, not significant; NUC, nucleos(t)ide analogue. ^†^ Model I included only baseline factors. Model II included both baseline and on-treatment factors.

**Table 4:** Univariate and multivariate analyses of factors associated with overall survival in subgroup patients with nucleos(t)ide analogue therapy.

		Univariate		Multivariate Model I ^†^		Multivariate Model II ^†^	
		HR (95% CI)	*p*	HR (95% CI)	*p*	HR (95% CI)	*p*
Baseline factors							
Age (years)	>60 vs. ≤60	0.872 (0.696–1.094)	0.236				
Sex	Female vs. male	0.901 (0.662–1.228)	0.510				
BCLC stage	C vs. B	2.544 (1.663–3.894)	<0.001	1.936 (1.233–3.041)	0.004	1.842 (1.149–2.953)	0.011
Portal vein invasion	Yes vs. no	1.623 (1.290–2.043)	<0.001		NS		NS
Vp4	Yes vs. no	1.423 (1.052–1.926)	0.022		NS		NS
Extrahepatic metastasis	Yes vs. no	1.224 (0.977–1.533)	0.079		NS		NS
AFP (ng/mL)	>400 vs. ≤400	1.778 (1.418–2.229)	<0.001	1.439 (1.127–1.837)	0.004	1.382 (1.033–1.849)	0.030
HBV DNA (IU/mL)	>20 vs. ≤20	1.593 (1.254–2.024)	<0.001		NS		NS
	>2000 vs. ≤2000	1.508 (1.173–1.938)	0.001		NS		NS
NUC starting time	Before TKI vs. After TKI	0.715 (0.535–0.957)	0.024	0.711 (0.521–0.971)	0.032		NS
ALBI grade	2–3 vs. 1	1.940 (1.534–2.452)	<0.001	1.655 (1.294–2.118)	<0.001	1.648 (1.239–2.193)	0.001
ALT (U/L)	>40 vs. ≤40	1.267 (1.012–1.586)	0.039		NS		NS
AST (U/L)	>40 vs. ≤40	1.480 (1.168–1.874)	0.001		NS		NS
Platelet count (10^9^/L)	>150 vs. ≤150	0.992 (0.792–1.241)	0.942				
On-treatment factors							
Achieving undetectable HBV DNA	Yes vs. no	0.496 (0.353–0.697)	<0.001			0.580 (0.411–0.817)	0.002
Second-line therapy	Yes vs. no	0.586 (0.465–0.737)	<0.001			0.620 (0.468–0.822)	0.001

Abbreviations: CI, confidence interval; HR, hazard ratio; NS, not significant; NUC, nucleos(t)ide analogue. ^†^ Model I included only baseline factors. Model II included both baseline and on-treatment factors.

## Data Availability

The data that support the findings of this study are available from the corresponding author upon reasonable request.
